# Carpooling Systems for Commuting among Teachers: An Expert Panel Analysis of Their Barriers and Incentives

**DOI:** 10.3390/ijerph19148533

**Published:** 2022-07-12

**Authors:** María del Carmen Rey-Merchán, Antonio López-Arquillos, Manuela Pires Rosa

**Affiliations:** 1 Consejería de Educacion y Deporte, Junta de Andalucía, 41018 Sevilla, Spain; mcrey@uma.es; 2 Economics and Business Management Department, University of Málaga, 29016 Malaga, Spain; 3 CinTurs—Research Center for Tourism Sustainability and Well-Being, University of Algarve, 8005-139 Faro, Portugal

**Keywords:** sustainable mobility, carpooling, smart mobility, sharing economy

## Abstract

Sustainable mobility is a current challenge in our society. Research shows that carpooling systems are potential solutions that could mitigate environmental pollution and urban congestion and provide cost savings for their users. Despite their potential benefits, the levels of carpooling practices among some occupations could be improved. Teachers are suitable for carpooling experiences due to their specific working conditions (e.g., timetables, destinations changes, path matches); however, there is no research solely focused on teachers. Thus, the current research aimed to analyze the barriers and incentives for teachers using carpooling systems for commuting. A panel member was selected following the staticized group technique. Panelists were surveyed to evaluate the advantages and disadvantages of carpooling. Results showed that fuel savings were considered by the expert panel as the most important incentive for carpooling. For short distances, carpooling was not considered the best commuting option. Additionally, the increase in travel time and loss of personal independence were identified as relevant barriers. Based on the opinions of experts, it can be concluded that carpooling barriers outweigh the incentives for the commuting of teachers. To promote carpooling practices, institutional mobility plans with advantages for carpoolers could improve the teachers’ perceptions about carpooling. Future carpooling strategies should consider these results to promote incentives and address the identified barriers.

## 1. Introduction

Transportation and mobility are key factors in societal development [1]. Sustainable economic and social development cannot be possible without sustainable transportation infrastructure and solutions [2]. The increased demand for safe, environmentally-friendly, and fast transportation of goods and individuals requires specific solutions [3].

In the European Union, institutions, enterprises, and governments are committed to increasing their knowledge, performance, and management of sustainable development. In this context, many communities are focused on limiting CO_2_ emissions through climate change adaptations and mitigation measures, to minimize the effects of CO_2_ emissions. For the success of the eco-friendly programs, profound changes are also required in the consumption patterns of citizens, as well as their participation in communities. Individual sustainability describes the potential of actions that enable and lead to attitudes and practices according to sustainability. The involvement of citizens, in an active way, in the sustainable development process, is the key to success [4].

Deep changes are needed for sustainable mobility, where the behaviors of citizens are determinant. Shared mobility, associated with the emerging ‘collaborative and sharing economy’ expressed in the European agenda [5], is one of the main citizen-centered contributions for smart and sustainable mobility. It contributes to a reduction in traffic congestion, decreased reliance on private vehicles, and lessens parking pressure in cities [6]. This innovative means of transportation has been mainly developed using technology to connect users and providers [7]. To promote sustainable solutions to mobility issues, some authors classified sustainable mobility measures on the basis of previous experiences [8], while other researchers studied travel patterns and psychological aspects associated with car-sharing [9].

One of the most simple examples of shared mobility is car-pooling, where people can offer free rides in their vehicles [10]. Research shows that the implementation of carpooling systems (by local authorities) is a potential solution that could mitigate environmental pollution and urban congestion [5]. Thus, a better understanding of drivers’ attitudes in practicing carpooling is necessary to promote the cited sustainable systems.

Carpooling can be defined as people—with similar origins and destinations—who share the same car (owned by one of the carpoolers) [11,12,13]. In car-sharing systems, users are not the owners of the car. They usually pick up a vehicle at one location and return it at any location [14], or they pay for a ride through an app [15].

Carpooling practices present several advantages from different perspectives. On the one hand, carpooling is an efficient way to reduce fuel consumption. In the 1970s, casual carpooling clubs (motivated by the oil crisis) were habitual in the USA [16]; however, a drop in oil prices contributes to the decline of carpooling [17]. The concern about pollution and congestion, and the continuous increase of oil prices in recent years, have contributed to an upward trend related to carpooling and car-sharing practices. In this sense, a previous study observed a direct relationship between carpooling and fuel price. It concluded that, in mainline lanes, traffic flow decreased when fuel prices increased, while carpool lane traffic flow increased [18].

Some authors have studied the impacts of this fuel reduction in different countries, such as the United States of America [19], China [20], and Iran [21]. The positive environmental effects of carpooling practices are complemented with other advantages, such as improved parking facilities [22], the reduction of congestion and traffic flow, and more efficient high-occupancy lanes [23]. Other positive effects of carpooling have been identified, such as increased productivity and morality of workers, and reduced stress from shared driving responsibilities [24]. However, psychological factors play important roles in adopting carpooling solutions [25,26,27,28]. Previous studies have identified some sociodemographic barriers, such as finding carpoolers with matching schedules, fear of sharing vehicles with strangers, and loss of freedom [29,30,31]. Other relevant barriers include extra travel times [32,33], distant meeting points [34,35,36], and loss of independence [37,38].

Commuting carpoolers will more likely address some of the cited potential problems [15,30,39], because colleagues share workplaces and timetables, and it is easier to trust people we know. In this sense, recent studies point out the importance of carpooling with a colleague, exhibiting the key role of trust [40]. Other authors have found large heterogeneities among solo commuting drivers [41], although they concluded that, for solo drivers, finding carpool drivers will be easier than carpool passengers.

Nowadays, the importance of carpooling programs has motivated the study of their effectiveness [42]. The cited authors propose a practical evaluation framework for a commuting carpool program [42].

In the particular case of Spanish teachers, their specific labor conditions provide good opportunities to practice carpooling. For the majority of teachers, they could change their habitual schools each year, if they agree to participate in an official procedure. In this procedure, teachers are ranked according to the length of service and some additional achievements. Based on these ranks, they can choose among the available destinations. Thus, once a year, many teachers are relocated according to the results of the cited procedure. According to that, the distance that a teacher must cover each academic year may vary. In addition, they share the same workplace with several colleagues, they have similar timetables, and in many cases, they match paths from their city of residence to their habitual workplace. However, the use of private cars by solo drivers is the most extended practice.

As previously described, several research studies can be found on the incentives and barriers of carpooling in the literature [43,44,45,46]. However, specific research studies were not found on teachers and their commuting trips. Due to the specific characteristics of this profession, a specific study that focused on teachers was of interest.

Thus, the current research aimed to analyze the barriers and incentives of carpooling systems (for teachers who commute). The paper is structured as follows. In Section 2, the methodology is described. The results of the panel member selections, questionnaire content, and data collection are presented in Section 3. Finally, we summarize the main findings and future research prospects in our conclusion.

## 2. Methodology

We used the Delphi Method (a single round), called the staticized group technique. The Delphi method is demonstrated to be an effective group-based judgment and decision-making method [47]. According to previous research based on the Delphi approach [48], the method can be defined as a systematic and interactive research technique used to obtain judgments from a panel of independent experts on a specific topic. Expert participants were selected according to predefined guidelines and they were required to participate in two or more rounds of structured surveys in a collaborative approach. After each round, an anonymized summary of participants’ results was provided. In each subsequent round, experts were encouraged to review the anonymous opinions of the rest of the experts and revise their previous inputs. The aim of this procedure was to decrease the variability of results and achieve a consensus about a correct value. Finally, the process was finished once the consensus was achieved or a number of predefined rounds were completed.

The staticized group technique is very similar to the Delphi Method. The only methodological difference is the absence of feedback or additional rounds in the staticized group technique. The Delphi methodology was carried out in fields, such as information systems [49] or worker safety [50]. Some studies have presented different opinions about the accuracy of consensus in the Delphi method. Some researchers did not report substantial differences in the accuracy of the results between Delphi and staticized approaches [51,52]. In contrast, other authors [53] promoted the use of the staticized group because participants were not led to achieve a consensus on a value that was not demonstrated as the best value. This was the main reason to use staticized groups in the current study.

It was not possible to generalize the results obtained from a small number of participants from a larger population (with statistical significance). However one of the main strengths of the staticized group methodology is that it takes advantage of the knowledge of experts in a specific topic. Experts selected were considered to have knowledge beyond a representative group. Then, the results obtained from the panel produced benefits for research and practice.

### 2.1. Panel Member Selection

In the methodology applied, expert selection is a key factor in the quality of the research. The majority of previous studies incorporated between 8 and 16 panelists and a minimum of 8 was suggested, while the authors suggested between 12 and 15 members [48]. The authors contacted 15 potential panelists from 4 different workplaces, in the region of Andalusia, in Spain. Finally, 12 experts completed the questionnaire provided on incentives and barriers to carpooling. Half of the selected experts were men (Experts 1 to 6) and the other half were women (Experts 7 to 12).

The level of expertise was defined by the previous authors [48] as the most important facet of a panel member. They proposed guidelines for the selection of experts with a flexible point system. Adaptation of the suggested point system to the specific objectives of our research is summarized in the following requirements, included in Table 1.

All members of the panel scored at least 30 points in addition to the 4 achievements (Table 2). Three other experts did not complete the questionnaire and they were excluded from the final results.

Regarding the personal conditions of the participants, the panel of experts was finally made up of 6 female professors and 6 male professors. Their ages ranged from 30 to 55 years. Ten of them were married with children, and only two were single. They all obtained a bachelor’s or baster’s degree before they started teaching.

### 2.2. Study Area and Potential Carpoolers

The region studied in the current research was Andalusia (Spain). It has a total area of 87.268 km^2^. Its extension is larger than some countries, such as Denmark, Holland, or Belgium, and is similar to Portugal. The number of teachers officially employed in Andalusia in the education sector (excluding universities) was higher than 125,000 in 2021. They daily commuted to one of the 3500 schools and high schools located in the region. Women represented 65% of teachers and men 35%.

Once a year, many teachers are relocated to a different school or high school; the distance that a teacher must commute varies. However, there are no official data available about their mobility patterns and transportation alternatives.

### 2.3. Questionnaire Content

The items selected for the questionnaire were chosen after a literature review focused on carpooling practices. In the first approach, 35 factors were considered. Then, the carpooling factors were classified as incentives or barriers. Finally, they were grouped and reduced to 20 items.

The following items were evaluated by the members of the expert panel. Items were classified into two main groups. In the first group, incentives for using the carpooler system were included (Table 3). The other group included the barriers to not participate in a carpooling system (Table 4). Additionally, items were classified by the following categories:

- Vehicle: includes the advantages and disadvantages related to the vehicle (such as fuel consumption or maintenance costs).
- Time: includes aspects related to the length of the commute (such as delay or length of displacement).
- Personal: individual factors, such as the genders of the carpoolers, or physical and mental fatigue.
- Social: human factors related to the social aspects of sharing a car.
- Environmental: reduction of emissions was considered in this category.

### 2.4. Conducting Phase-Data Collection

A digital survey was used to collect the participants’ opinions. The following strategies were integrated into the survey to reduce the bias and to improve the quality of the research:

- The order of the questions was randomized for each participant to reduce the contrast effect and primacy effect.
- The anonymity of each panel member was ensured.

A Likert scale was proposed to the experts to evaluate the items. The Likert scale can be defined as a psychometric tool with a set of statements of the study’s hypothesis [67]. Panelists were asked to state their levels of agreement with the given statements (from strongly agree to strongly disagree). The length of the Likert scale could be designed with different measurement ranges in terms of the number of response options (from two to eleven points) [68]. Despite shorter rating scales being quick to use, scales with 10 and 11 alternatives were preferred to express respondents’ feelings adequately. In this sense, some authors concluded that 10-point, 9-point, and 7-point scales are the most preferred rating scales [69]. In the current research, a 10-point scale was used to collect opinions from the expert panel.

## 3. Results and Discussion

Different benefits and barriers of carpooling practices have been evaluated in the research. Some of them were previously studied in the literature. A consensus was not required for the staticized group methodology; variance and standard deviations were calculated to compare the results obtained with a Delphi method approach with additional rounds of feedback. On the one hand, all incentive items obtained values under 10% variance and deviation. On the other hand, some barrier items did not reach consensus values under the cited 10%. Thus, it can be said that consensus is lower regarding the barriers to adopting carpooling. Questionnaire reliability was calculated with Cronbach’s alpha values. Items classified as incentives obtained a Cronbach’s alpha of 0.67. This value is considered a satisfactory level of consistency [70]. For the group of barriers, Cronbach’s alpha value was 0.83, which is considered a high level of consistency [70]. Then, the incentives and barrier questionnaires obtained reasonable and accepted values of reliability.

### 3.1. Incentives for Carpooling

One of the most studied incentives is the impact of cost-saving (regarding attitude toward carpooling) [24,37,38,44,54,55]. Our results (Table 5) showed that the expert panel considered fuel saving to be the most important incentive to carpooling (AVG = 9.83; VAR = 0.31; DESV = 0.58). Similarly, other authors identified saving money as the main advantage perceived by users [37,44]. Commuting cost burden is not a new reason for carpooling—it was highlighted by previous researchers a long time ago [71,72]. Current research identifies cost-saving as the most important encouraging factor for carpooling [43]. In the cited research, 46.1% of current carpoolers note that carpooling is less expensive than driving alone. Potential carpoolers showed lower scores in this factor (39.5%). Aligned with the cited results, in research carried out in Sweden based on carpooling experiences, it was concluded that 43.4% of participants stated cost as the most important motive to carpool [73]. It can be observed that percentages associated with cost savings are similar in different countries.

Different types of costs associated with the use of the vehicle, such as fuel, parking, or maintenance, were similarly rated in our research. The difference between fuel-saving and maintenance saving (AVG = 9.58; VAR = 0.41; DESV = 0.67) was very low. Regarding cost savings (fuel and maintenance), there were no significant differences between male and female opinions. In the literature, the differences were not significant. Cost of parking (41.3%) and cost of fuel (40.1%) were noted as relevant factors by habitual carpoolers [43]. Aligned with these findings, other research based on the opinions of usual carpoolers in San Francisco Bay (USA) concluded that the most important reason for casual carpooling was monetary savings (40%) [19].

The impact of fuel-saving decreases when the commuting distance is low [54]. In the same way, carpooling is more likely to be considered when the costs of solo-driving are high [46]. Then, other factors can increase their importance. Moreover, it should be considered that some variations in fuel costs, or the introduction of electric cars, could change the importance of fuel-saving as an incentive.

The group of personal factors was scored as the second group of highest values. Mental and physical fatigue reduction were scored with values near to 8 and over 10 in the Likert scale. Aligned with that results, previous researches revealed a positive relationship between carpooling and well-being during commuting [56]. Similarly, other researchers highlighted stress reduction as an important benefit associated with carpooling experiences [24]. Physical and mental benefits are difficult to quantify, but their positive effects should not be undervalued.

Regarding the social advantages of carpooling, they obtained values over 7, but they were the lowest-rated group of advantages when compared with the rest of the items. Then, social advantages were the less important group in the sector studied. Similarly, other research found that socializing was the least popular motive to carpool. Only 5.7% of the participants noted that reason as an incentive [73].

Finally, environmental reasons, such as a reduction in emissions, obtained an average value of 8.08. Although the value could be considered high, it was lower than fuel saving (9.83). Results associated with environmental concerns varied. On the one hand, previous research ranked low environmental reasons for carpooling [19]. On the other hand, other authors [73] found that 35.2% of users stated environmental concerns as the reason for carpooling. To increase the knowledge about the environmental benefits of carpooling, the design and use of indicators that inform the users of the polluting gases not emitted due to carpooling are recommended.

### 3.2. Barriers to Carpooling

Once the perceptions of incentives were analyzed, the next step was to analyze the barriers to adopting carpooling with the aim to understand the low levels of carpooling practices in the sector.

According to the experts (Table 6), the main barrier to adopting carpooling for commuting was low fuel saving (AVG = 9.92; VAR = 0.08; DESV = 0.29). This value was aligned with the results obtained from the incentives related to fuel consumption. Previous studies concluded that sharing the costs makes carpooling economically competitive compared to solo driving, at least in long distances [45,57]. As a consequence, for short displacements with low fuel cost savings, carpooling is not considered the best commuting option [54]. However, these costs can be underestimated by the user, since the daily sum of short journeys represents an accumulated expense throughout the year that could make the user change his/her mind. A carpooling platform that estimates the annual savings based on the length of the journey could be of interest to eliminate this barrier.

Spending time waiting for workmates (AVG = 8.00; VAR = 1.00; DESV = 1.04) and a bigger probability of delay (AVG = 8.08; VAR = 0.58; DESV = 0.79) were identified as second and third barriers in weight. In research carried out in the USA [43], it was found that more than half of the participants stated that they did not have time to wait on others. This barrier obtained similar values among users, potential users, and people not interested in carpooling.

The increase in travel time and waiting time were previously noted as important disadvantages to carpooling [32]. Some authors found that the value in travel time in carpooling is more than twice for solo driving for long distances [45]. Other authors found a slightly higher value in travel time for carpool modes than for solo driving [33]. The majority of studies noted that carpooling increased travel time.

Another relevant barrier in the opinions of experts was the need for displacement to the meeting points (AVG = 7.75; VAR = 0.52; DESV = 0.75). This problem was addressed in previous studies [35,36]. They considered meeting points or hubs as possible solutions in the design of carpooling systems (instead of barriers) and some authors concluded that adding the option of meeting hubs improves system-wide savings from carpooling. However, it is important to place meeting points properly, because some authors identified distant meeting points as one of the most important barriers to adopting carpooling [34].

In real-world carpooling, an extra displacement to the hub is considered a disadvantage by carpoolers when they decide between carpooling or solo-driving; however, optimized hubs can promote carpooling practices [35]. A higher number of potential users could provide the best matching rates to reduce the need for meeting points.

Regarding personal variables, they scored relevant values. Loss of personal independence to travel was considered an important barrier. The results are aligned with previous studies [37,38]. In a previous carpooling experience carried out in New Zealand, users noted that being dependent on other users’ timetables and the lack of flexibility as relevant disadvantages [46]. Low matches in working tables obtained low values among the expert participants. Aligned with that opinion, the variety of pick-off and drop-off times obtained low values among habitual users (25.7%) [43]. In contrast, potential carpoolers were more concerned with the timetables (43%).

Finally, the group of social barriers obtained the lowest values of importance, but the higher values of variance and deviation. In the case of social awkwardness (AVG = 4.00; VAR = 2.83; DESV = 1.76), the low values can be motivated because teaching is a team task, and teachers are in constant communication with their colleagues. Therefore, most of them know each other. This factor contributes to increased trust in potential carpooler mates [15,39]. The higher values of variance and deviation were motivated by the differences between male and female scores. In previous studies, females were found to be less attracted to carpooling [33,66,74], possibly due to security concerns. A higher number of potential users could provide the best matching rates according to the gender preferences of the carpoolers.

## 4. Conclusions

Based on the results obtained, it can be concluded that the barriers to carpooling outweigh the incentives for teachers to travel. Most of the specific results obtained for teachers were aligned with previous general results obtained by other authors. Despite the specific working conditions of teachers, their attitudes toward carpooling were not particularly different compared to other groups of people previously studied, such as students and industry workers.

Cost savings related to fuel consumption were identified as the main incentives (or advantages) of practicing carpooling. However, the importance (or weight) of these incentives decrease if the length of the displacement is short because cost savings are poor.

Psycho-social factors related to personal and social variables were regarded as obstacles to performing carpooling practices. These barriers were difficult to change according to the existing literature. The most effective way to reduce the weight of these disadvantages is to increase the efficiency of carpooling systems because the weight of cost savings will increase too.

In the group of female experts, the items related to barriers obtained higher results than the group of male participants. Females placed different genders of coworkers as the more relevant barrier compared to males for the same item. A possible solution to reduce this barrier could be the management of some vehicles exclusively for women. This solution would allow the group of women with less confidence to share their cars in conditions that are desirable for them. In this sense, some previous experience based on “pink transportation” has been developed in some countries [75].

In contrast, male and female panelists scored similar values regarding the incentives for carpooling.

According to the results obtained, the regional government could develop a carpooling system for teachers in Andalusia based on the main incentives found. An institutional carpooling program would increase the number of possible combinations to carpooling among users and improve their matching possibilities. This could reduce some barriers, such as the need for displacements to meeting points, earlier beginnings of journeys, and the genders of coworkers.

Additionally, a formal carpooling system with real-time information about cost savings and emission reductions could encourage potential carpoolers to carpool.

Future carpooling strategies should consider the results achieved to promote incentives and address the identified barriers.

### 4.1. Limitation of the Study

The current research was focused on teachers from a specific region of Spain. Different labor conditions for teachers in other regions or countries might change the opinion of experts. Similarly, different cultures, safety levels, or social habits could change the opinions of potential carpooling users.

### 4.2. Future Research

In future research, a combination of experts from different countries could increase the scope of the results.

The design and assessment of a real carpooling system by the same group of experts combined with a questionnaire for users could improve their knowledge about the real problems of carpooling systems and create new solutions to mitigate existing barriers.

## Figures and Tables

**Table 1:** Flexible point system for teacher selection.

Achievements or Experience	Code	Points
Teacher who drives to commute	A1	4
Year of experience as a carpooler	A2	3
Change in his/her work destination	A3	2
Habitual workplace in a different city	A4	3

**Table 2:** Expert scores in the flexible point system.

Expert	A1	A2	A3	A4	Total
1	4	27	4	3	38
2	4	30	2	3	39
3	4	45	2	3	54
4	4	51	6	3	64
5	4	36	4	3	47
6	4	36	2	3	45
7	4	21	4	3	32
8	4	45	6	3	58
9	4	33	8	3	48
10	4	54	8	3	69
11	4	42	6	3	55
12	4	30	2	3	39
AVG	4	38	5	3	49
MED	4	36	4	3	48
DESV	0	10.03	2.28	0	11.27

**Table 3:** Incentives to adopting carpooling for commuting.

Category	Incentives	Authors	Code
Vehicle	Fuel-saving	[24,37,54,55]	I1
	Vehicle maintenance saving	[37,54]	I2
Personal	Reduce driving physical fatigue	[37,44,56]	I3
	Reduce driving mental fatigue	[37,44,56]	I4
	Rest during the displacement	[37,44,56]	I5
Social	Support from coworkers in the same situation	[38,44]	I6
	Improves relations between coworkers	[32]	I7
	Socialize out of the worksite	[57]	I8
	Better communication with coworkers	[58,59]	I9
Environmental	Reduce emissions	[19,31,60]	I10

**Table 4:** Barriers to adopting carpooling for commuting.

Category	Barriers	Authors	Code
Vehicle	Low fuel-saving	[45,57]	B1
Time	Spending time waiting for coworkers	[32,33,45]	B2
	More probability of delay	[32,33,45]	B3
	Earlier start of the journey	[58]	B4
	Need of displacement (regarding the meeting points)	[61]	B5
Personal	Less Independence	[37,44]	B6
	Different genders of coworkers	[25,30,62]	B7
Social	Social awkwardness	[27,63]	B8
	Extending work problems to the car	[64,65]	B9
	Low-match of coworkers’ timetables	[66]	B10

**Table 5:** Evaluation of incentives for carpooling from panelists.

Expert	I1	I2	I3	I4	I5	I6	I7	I8	I9	I10	Total
1	10	10	8	7	7	7	6	6	7	9	77
2	10	10	9	9	8	8	8	8	8	8	86
3	10	10	8	8	8	8	8	7	7	8	82
4	10	10	9	9	9	8	7	7	7	8	84
5	10	10	8	8	8	7	6	6	7	9	79
6	10	9	7	7	7	7	7	8	7	8	77
7	10	9	7	8	7	7	7	7	8	8	78
8	10	10	8	8	8	8	7	6	8	9	82
9	10	10	8	8	8	8	7	7	7	7	80
10	10	9	9	9	9	8	8	8	8	8	86
11	10	10	7	7	7	7	7	8	7	8	78
12	8	8	7	7	7	8	9	8	8	7	77
AVG	9.83	9.58	7.92	7.92	7.75	7.58	7.25	7.17	7.42	8.08	AVG
VAR	0.31	0.41	0.58	0.58	0.52	0.24	0.69	0.64	0.24	0.41	VAR
DESV	0.58	0.67	0.79	0.79	0.75	0.51	0.87	0.83	0.51	0.67	DESV

**Table 6:** Evaluation of the barriers to carpooling from panelists.

Expert	B1	B2	B3	B4	B5	B6	B7	B8	B9	B10	Total
1	10	9	9	8	9	7	6	7	3	7	68
2	10	9	9	7	9	8	5	7	4	8	68
3	10	8	8	6	8	8	5	5	5	9	63
4	10	10	9	7	8	9	4	5	4	8	66
5	10	8	7	6	7	8	5	4	3	7	58
6	10	8	8	8	8	7	6	5	3	7	63
7	10	8	9	7	9	8	3	4	3	8	61
8	10	7	8	8	6	7	2	4	4	8	56
9	10	8	8	7	8	8	2	3	5	8	59
10	10	8	8	6	7	8	6	6	2	8	61
11	10	6	7	7	8	6	1	2	4	6	51
12	9	7	7	5	5	5	3	2	1	6	44
AVG	9.92	8.00	8.08	6.83	7.67	7.42	7.50	4.00	4.50	3.42	AVG
VAR	0.08	1.00	0.58	0.81	1.39	1.08	0.75	2.83	2.58	1.24	VAR
DESV	0.29	1.04	0.79	0.94	1.23	1.08	0.90	1.76	1.68	1.16	DESV

## Data Availability

Not applicable.

## Abbreviations

The following abbreviations are used in this manuscript:

A	achievement
AVG	average
B	barrier
DES	deviation
I	incentive
MED	median
VAR	variance
